# Evaluation of Microchip-Based Point-Of-Care Device “Gazelle” for Diagnosis of Sickle Cell Disease in India

**DOI:** 10.3389/fmed.2021.639208

**Published:** 2021-10-13

**Authors:** Shweta Shrivas, Madhav Patel, Rajat Kumar, Anil Gwal, Ramswaroop Uikey, Shashi Kant Tiwari, Anil Kumar Verma, Priyaleela Thota, Aparup Das, Praveen Kumar Bharti, Rajasubramaniam Shanmugam

**Affiliations:** ^1^ICMR-National Institute of Research in Tribal Health (NIRTH), Jabalpur, India; ^2^Hemex Health, Portland, OR, United States

**Keywords:** hemoglobinopathies, sickle cell disease, thalassemia, India, point of care (POC) diagnosis

## Abstract

Sickle cell disease is a major public health problem in India. Lack of rapid and reliable diagnostic methods result in many avoidable deaths in affected population. Current diagnostic tools are laboratory based, expensive and need trained manpower. Here, we evaluated the performance of a microchip-based cellulose acetate electrophoresis test, “Gazelle” in the tribal-dominated Indian states of Chhattisgarh and Madhya Pradesh. A total of 1,050 patients were screened by sickle cell solubility, hemoglobin (cellulose acetate) electrophoresis, high-performance liquid chromatography (HPLC) and Gazelle. Of the total 1,027 test results obtained, 960 tests were “Valid” (93.5%) and included in the analysis. Gazelle identified all patients with disease (HbSS and Thalassemia Major) with 100% accuracy. Gazelle demonstrated 100% sensitivity when comparing sickle cell disease (SCD) vs. sickle cell trait and SCD vs. normal. Specificity was 98.9% and 99.5% when comparing SCD vs. trait and trait vs. normal, respectively. Specificity was 99.8% when comparing SCD vs. normal and sensitivity was 99.3% when comparing trait vs. normal. Overall, Gazelle yielded a high accuracy (99.0%) compared to reference standard tests (hemoglobin electrophoresis and HPLC). Gazelle is a low-cost, rapid diagnostic test with high accuracy for detecting SCD both quantitatively and qualitatively. Gazelle can be a potential screening tool for the rapid diagnosis in resource limited settings and developing countries with high burden of hemoglobin disorders.

## Introduction

Hemoglobinopathies are the most common autosomal hereditary disorders. Approximately 7% of the global population carries hemoglobin gene mutation including structural hemoglobin variants like sickle hemoglobin or thalassemia (1–3). Hemoglobin S is highly prevalent in sub-Saharan Africa (4), Mediterranean region, India and Southeast Asia (5). India alone contributes about 15% of all sickle cell anemia (SCA) newborns worldwide (6). An estimated 20% children with sickle disease die in India before the age of 2 years (7) and 30% of tribal children with sickle cell disease (SCD) die before the age of 5 years (8). Prevalence of SCA is predominantly found among 3 socio-economically disadvantaged ethnic groups; the Scheduled Tribes, the Scheduled Castes and Other Backward Class communities in India (9). Jagdalpur, Chhattisgarh is a tribal dominated district in India and is mirrored in the fact that a majority of the patients belong to Scheduled tribe communities (58.7%) followed by Scheduled castes (18.7%) and Other Backward classes (17.9%). Tribal populations constitute 8.6% of the total Indian population and the prevalence of sickle cell carriers and SCD among these different tribal groups varies from 1 to 40% and 1to 2%, respectively (10).

Hemoglobin S is a structural variant of normal adult hemoglobin caused by replacement of glutamic acid by valine at position 6 of beta-globin (11–13). This mutation causes polymerization of HbS in low oxygen conditions which can distort red blood cells into a sickle, or crescent, shape. Recurrent sickling episodes lead to RBC lysis. Sickle cell anemia is characterized by pain crisis, severe anemia, jaundice, infarction, stroke, etc. resulting in high morbidity and mortality (14).

Heavy morbidity and mortality among rural and tribal populations is mainly due to lack of diagnosis. Early diagnosis is crucial to initiating life-saving therapies and knowledge of sickle cell carrier status is critical to prevention and parental planning for at-risk populations. Currently, only a few public health facilities for diagnosis of SCD and patient care exist. Moreover, with no universal screening program in place, the overall burden of SCA remains unidentified. Although, in 2015, the Govt of India initiated a program for screening SCA among tribal school children, only a few states have implemented this program. In this program, low cost sickle solubility test was used due to the absence of high-performance liquid chromatography (HPLC) and hemoglobin electrophoresis. These gold standard tests require advanced laboratory facilities, trained staff and are slow and have high input costs. Though sickle solubility and sickling slide tests are popular field tests and are considered to have high sensitivity, they cannot differentiate between carrier and disease status (15). In addition, the limit of detection of solubility tests depend on Hb percentage, coinheritance of alpha-thalassemia, hereditary persistence of fetal hemoglobin, increased serum viscosity, hyperlipidemia, elevated serum proteins, etc. that may result in false negative or false positive test results. On the other hand, sickle-slide test has fewer limitations, but requires a light microscope, longer incubation time, and trained microscopists. Poor slide preparations result in false negatives. In this context, there is an urgent need for an affordable, portable, easy-to-use, accurate, point-of-care tests for SCA testing in resource-limited countries.

**Figure 1:**
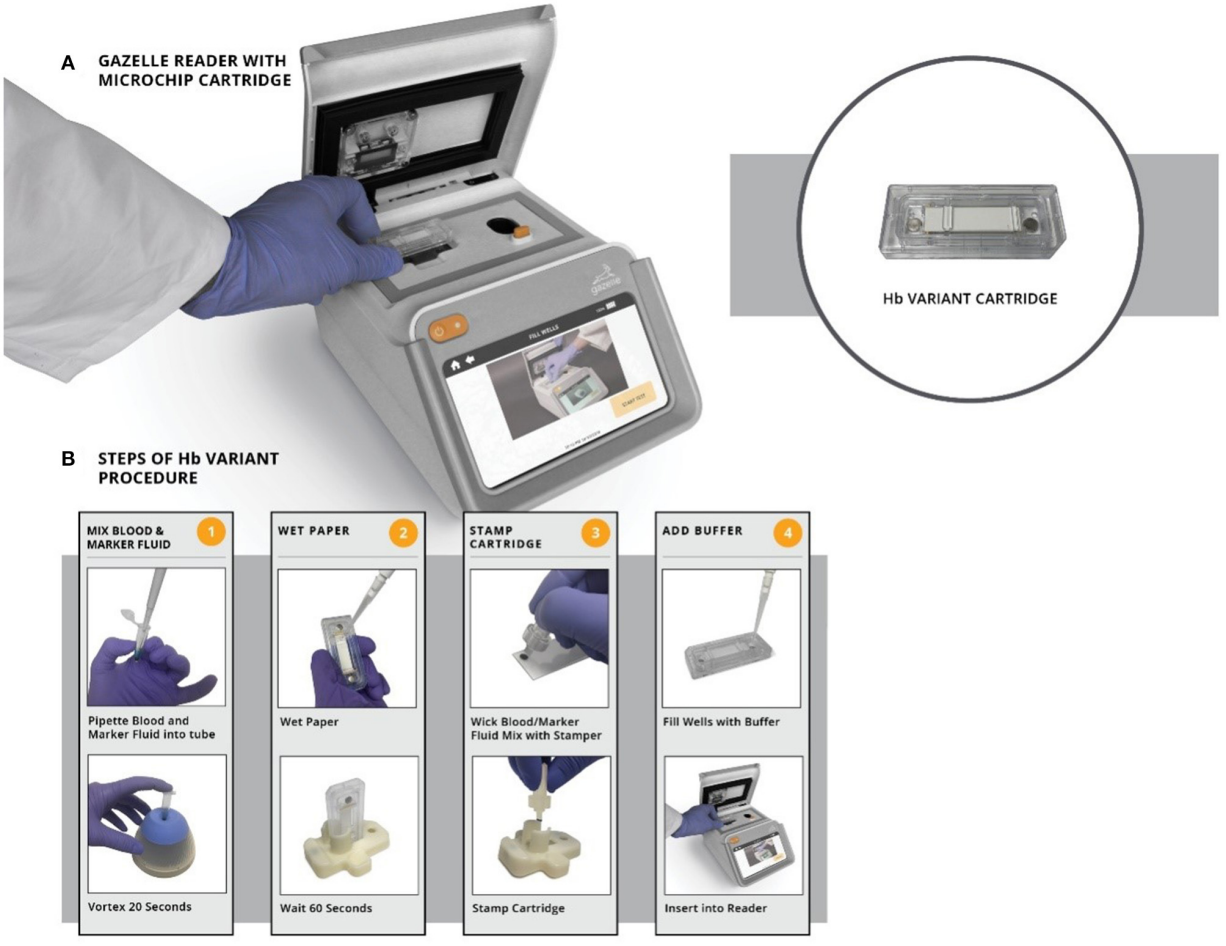
Gazelle-microchip electrophoresis: **(A)** Gazelle Reader with Cartridge. **(B)** Steps of Gazelle Hb Variant Procedure.

“Gazelle” (Figure 1) is a portable microchip electrophoresis platform that includes a reader and cellulose acetate cartridge for identifying the most common hemoglobin variants at the point-of-care in low-resource settings (16). Here, we report the diagnostic performance results from a field study of Gazelle.

## Materials and Methods

The study was conducted at the sickle cell clinic of ICMR-National Institute of Research in Tribal Health (NIRTH) at Late Baliram Kashyap Medical college, Jagdalpur, Chhattisgarh during August 2018 and November 2019. The study protocol was approved by the Institutional Ethical Committee of ICMR- National Institute of Research in Tribal Health, Jabalpur, India (NIRTH/IEC/1153/2017) and Health Ministry's Screening Committee (HMSC) ICMR, India. Informed consents were obtained from all study participants. The study was supported by Hemex Health, Portland, Oregon, United States of America and one of authors is affiliated to the company.

### Gazelle

Details of the Gazelle (HemeChip) design have been previously published (16). Briefly, Gazelle is a single-use cartridge-based test that enables rapid, affordable, quantitative, accurate diagnosis for both SCA and carrier status at the point of care. It is a cellulose acetate-based microchip electrophoresis system within a portable instrument, utilizing the principle of standard electrophoresis method with inbuilt analysis software, electronic data storage, and wireless data transmission capabilities. The device is operated by rechargeable lithium batteries that can test all day on a single charge. The device also carries advanced features like WiFi, GPS, and Bluetooth enabled connectivity for easy tracking of samples and can connect to a printer wirelessly.

### Study Design and Participants

Clinical study design, study participants, sample size calculation, and details on test methods are described according to the Standards for Reporting Diagnostic Accuracy (STARD) guidelines (17).

Based on preliminary studies with HemeChip (pre-Gazelle prototype) (16), an evaluation of 1000 samples with Gazelle was planned. Blood samples were collected from patients visiting Late Baliram Kashyap Medical College, Jagdalpur, Chhattisgarh either for treatment of anemia or the antenatal clinic. Inclusion criteria included: subjects of age 6 months to 65 years with or without the signs and symptoms of pallor, jaundice, abdominal pain, joint pain and all pregnant women. A signed informed consent (either by the subject or by the parent as appropriate if the subject is a minor) was required for study inclusion. Subjects with a history of blood transfusion in the previous 3 months and those who withdrew their consent after enrolling were excluded from the study.

No incentives were provided to the subjects or staff for participating in the study. Patients who participated in the study received medical care according to standard procedures of the site. Test results from Gazelle were not made available or used for medical care.

### Blood Sample Acquisition and Testing

From each subject, 2 mL of peripheral blood were collected in a vacutainer containing EDTA as an anticoagulant. All samples were tested by sickle solubility test (18), standard laboratory-based reference standard cellulose acetate electrophoresis (19) and Gazelle. Sickle positive samples were also confirmed by HPLC using betathal short program (VARIANT™ II, Bio-Rad Laboratories, Inc., Hercules, California, USA). HPLC and standard cellulose acetate electrophoresis were considered to be the reference standard for our comparisons.

Clinical information and reference test results were not available to the performers of the test or the study team at the time of testing. Similarly, Gazelle test results were not available to the performers of the standard reference tests. Sickle solubility test, cellulose acetate hemoglobin electrophoresis, complete blood count and Gazelle testing were carried out at the sickle cell clinic of ICMR-National Institute of Research in Tribal Health, Jagdalpur, Chhattisgarh. Per protocol, HPLC testing was planned for at least one-third samples testing positive for SCD or SCD Trait. HPLC was performed at ICMR-NIRTH Laboratory at Jabalpur.

**Figure 2:**
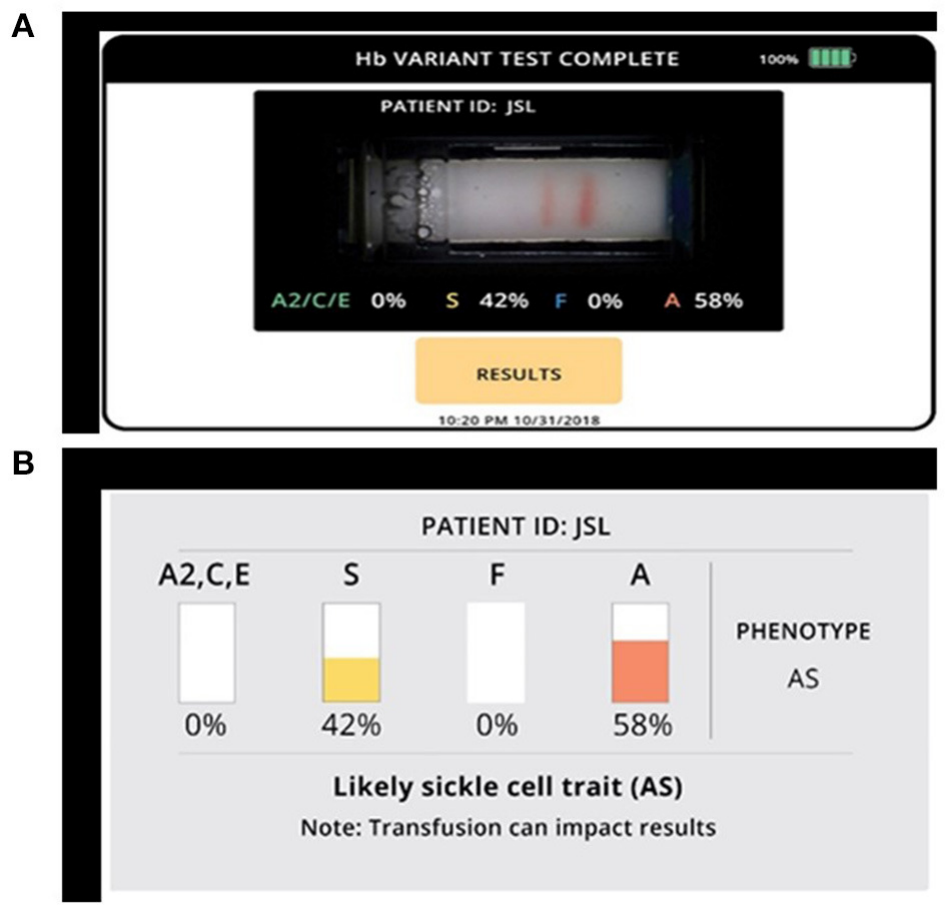
Gazelle shows qualitative and quantitative measurements of various hemoglobin variants. **(A)** Microchip electrophoretic image showing separation of HbA and HbS with per cent quantification. **(B)** Diagrammatic representation of various hemoglobin variants.

Samples for Gazelle were processed as per manufacturer's instructions (Figure 1). Briefly, 20 μL blood was mixed with 40 μL lysing solution. The cellulose acetate membrane inside the Gazelle cartridge was pre-wetted with TBE buffer (50 μl at pH of 8.4) and lysed blood sample stamped with a stamper. Next, 200 μl TBE buffer was added to both buffer wells and the cartridge was placed in the Reader and charge was applied. Migration and separation of various bands was completed in 8 min. At this end point, the bands were automatically identified by the inbuilt custom software in the reader. Results on Gazelle are easy to read as the identity and quantity of different Hb variants are displayed on the reader (Figures 2A,B).

### Statistical Methods

Diagnostic accuracy of Gazelle in comparison to hemoglobin electrophoresis was determined as the percent ratio of correct tests results, and summation of correct tests results and incorrect test results for a hemoglobin variant category. Sensitivity was determined as the ratio of true positive results divided by the summation of true positive results and false negative results. Specificity was determined as the ratio of true negative results divided by the summation of true negative results and false positive results.

## Results

### Participants

Overall, 1,050 subjects participated in this study. The average age of participants was 22.4 years. The majority of the participants were from Scheduled Tribe communities (617/1050; 58.7%), followed by Scheduled Caste (197/1,050; 18.76%), Other Backward caste (OBC; 188/1,050; 17.9%) and General caste (48/1,050; 4.6%). Of these, 259 (24.7%) participants were male and rest were female. A total of 1,050 samples were collected and screened for SCA by sickle solubility, hemoglobin (cellulose acetate) electrophoresis and Gazelle. HPLC analysis was also performed on a subset of 186 samples.

### Gazelle Performance

A total of 960 samples were included in the analysis; 23 samples were not included in the analysis: data files were not received due to communication error for 16 samples and seven samples due to probable sample mix ups.

Of the total 1,027 test results obtained, 960 tests were “Valid” (93.5%) and included in the analysis, 41 tests were “Uninterpretable” (4.0%), and 26 tests were “Inconclusive” (2.5%) (Table 1). An “Inconclusive” or “Uninterpretable” test does not result in a diagnostic decision. As such, when an “Inconclusive” or “Uninterpretable” test is encountered, the test can be repeated or other methods of testing can be utilized, which are common practices in diagnostics, as well as in hemoglobin testing. Therefore, based on recommended practices in the literature and the STARD guidelines, the inconclusive and uninterpretable test results were reported separately in this study (Table 1).

**Table 1:** Overview of diagnostic tests with Gazelle.

	**Total**	**Valid tests**	**Uninterpretable tests**	**Inconclusive tests**
Subjects and tests	1,027	960	41	26
Percentage	100	93.5	4.0%	2.5%

**Figure 3:**
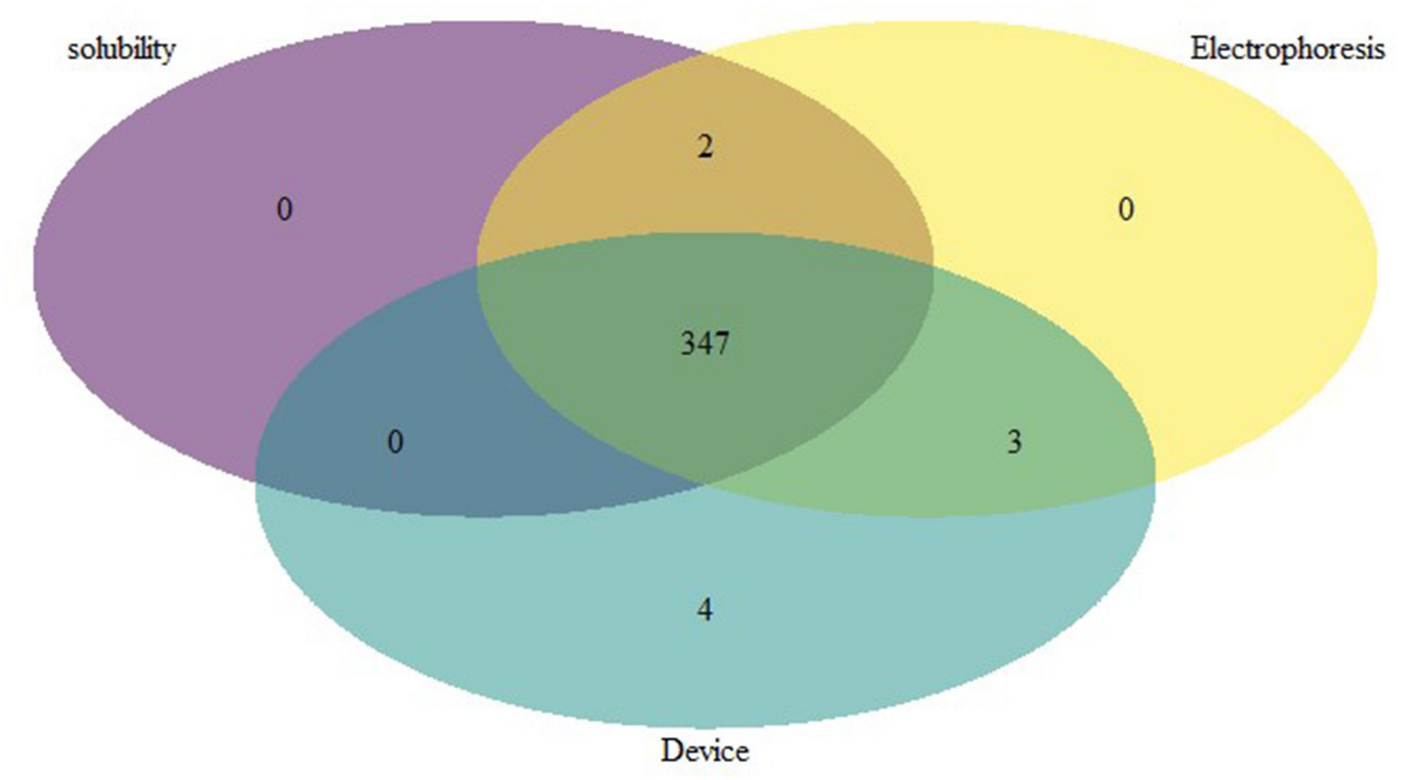
Venn diagram depicting the comparative performance of different sickle diagnostic tests. The overlap result of diagnostic test showed that 347 samples with HbS were found to be positive in all 3 different tests.

In the analysis, 65 samples were positive for sickle cell anemia by electrophoresis, solubility, and device, 347 samples were positive for Sickle cell trait by all 3 methods (Figure 3) and 604 samples were normal (HbAA) by all methods (Table 2). Gazelle test results from this study included SCD-SS (Hb SS), SCD Trait (Hb AS), Hb E Trait (Hb AE), Beta Thalassemia Major and Normal (no abnormal Hb, or Hb AA). Gazelle identified subjects with disease, SCD-SS with 100% accuracy (Table 2). Two Beta Thalassemia Major subjects were identified as Hb SS disease by Gazelle. Two samples with traits (Hb AS) were identified as normal (Hb AA) due to light S bands, 2 were identified as SS and one was identified as SF by Gazelle. Three subjects with no abnormal Hb (Hb AA) were identified as AS due to smearing of the bands and one was identified as SS by Gazelle (Table 2). Gazelle yielded an overall high accuracy (99.0%) compared to reference standard tests (HPLC and hemoglobin electrophoresis).

**Table 2:** Diagnostic accuracy of Gazelle compared to hemoglobin electrophoresis.

**Category**	**Hemoglobin type**	**Correct**	**Incorrect**	**Accuracy**
Sickle cell disease, SCD-SS	HbSS/HbSF	67	0	100%
Beta thalassemia major	HbF	0	2	0%
SCD trait	HbAS	277^*^	5	98.2%
Hb E trait	HbAE	1	0	100%
Normal (no abnormal Hb)	HbAA	604	4	99.3%
All categories	–	949	11	98.9%

* *1 AS trait called AC or AE trait by Gazelle – considered correct*.

Sensitivity and negative predictive value (NPV) were 100% for SCD-SS vs. other hemoglobin variants (Table 3). Specificity was 99.3% for SCD-SS vs. others, and 99.6% for SCD Trait vs. others (Table 3). Positive predictive value was 91.8% for SCD-SS vs. others, and 98.9% for all SCD Trait vs. other types (Table 3). Gazelle showed 100% sensitivity when comparing disease vs. trait and disease vs. normal. Specificity was 98.9% and 99.5% when comparing disease vs. trait and trait vs. normal, respectively. Specificity was 99.8% when comparing disease vs. normal and sensitivity was 99.3% when comparing trait vs. normal.

**Table 3:** Gazelle diagnostic sensitivity, specificity, positive predictive value (PPV), and negative predictive value (NPV) in comparison to reference standard method.

	**SCD-SS vs others**	**SCD Trait vs others**
True positive	67	277
True negative	887	675
False positive	6	3
False negative	0	5
Sensitivity, TP/(TP+FN)	100%	98.2%
Specificity, TN/(TN+FP)	99.3%	99.6%
PPV, TP/(TP+FP)	91.8%	98.9%
NPV, TN/(TN+FN)	100%	99.3%

**Figure 4:**
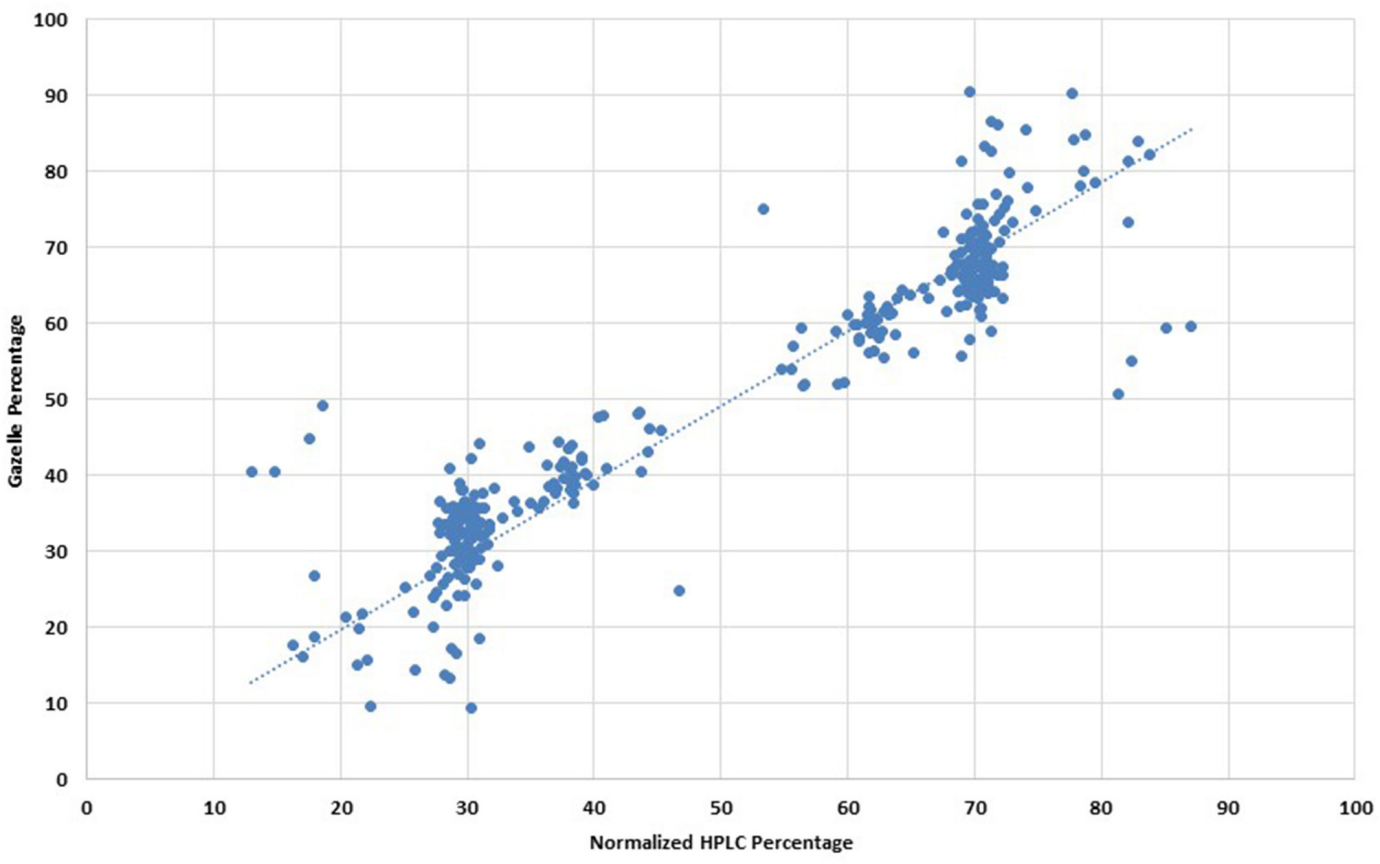
Comparison of hemoglobin quantification percentages between normalized HPLC and Gazelle. *Note: This is applicable to all Hb types, not only HbS. Graphic representation of Pearson Correlation Coffeficient analysis.

For clinical studies that report diagnostic accuracy, published guidelines recommend excluding inconclusive results from sensitivity and specificity analyses, instead, an additional summary statistic can be reported that accounts for inconclusive results (16). Gazelle yielded a diagnostic accuracy of 93.5% in the additional analysis wherein inconclusive tests were considered as incorrect. Quantification for 161 multi-band tests with the same phenotype between Gazelle and HPLC demonstrated a Pearson correlation coefficient of 93.3% (Figure 4). No adverse events were reported during the study. False-negative sickle-solubility test reactions are known to occur with insufficient quantities of blood from anemic patients (hemoglobin levels <8.0 g/dL). Four hundred and fifty-one samples had Hb levels lower than 8.0 g/dL and in such cases minimum 2 volumes (40 μL) were used for solubility test. In contrast, Gazelle could detect presence of HbS even in samples where Hb levels were lower than 4.0 g/dL without increasing input volume (data not shown). In all, 152 samples had Hb levels lower than 4.0 g/dL.

## Discussion

In this point-of-care field study conducted in a remote tribal area, Gazelle exhibited high sensitivity and specificity for detection of Hb types, HbA, HbS, and HbF, and high sensitivity and specificity for each relevant Hb phenotype (normal, carrier, and disease states). Notably, all subjects with SCD were identified with Gazelle.

Despite their inability to differentiate between carrier and disease status, solubility and sickling slide tests are popular field tests that are often employed for sickle cell diagnosis (15). Follow-on confirmatory tests such as HPLC or hemoglobin electrophoresis are needed and these tests require advanced laboratories (20). Furthermore, advanced laboratory techniques require trained personnel which are lacking or in short supply in underdeveloped and developing countries, a situation which is more acute in areas of high prevalence of hemoglobin disorder. Gazelle is a low cost, rugged device designed for low resource settings. It has a built-in software that detects errors occurring during sample preparation or testing process and calls automatic retests. It has an automated analysis system to perform interpretation of each sample. This feature has been shown to limit user errors in pilot studies and is also supported by Gazelle's extremely low frequency of discordant results in this study. The short test time allows testing while the patient waits to ensure that test results reach the patient and clinical intervention, if needed, can start immediately. One other Gazelle feature that limit user errors and the time needed for training is the included step by step instructions displayed on the screen to guide the user during the testing process. Also, customized reports can be generated from the device and with the traceability of reports and data, clinicians or laboratories can maintain a record of patient's history. Test results can be stored on the reader, saved in the Cloud or printed.

In this study, Gazelle, a new point-of-care, microchip-electrophoresis based sickle diagnostic device was compared to conventional laboratory-based hemoglobin electrophoresis and HPLC. The sensitivity and specificity of the Gazelle is comparable to hemoglobin electrophoresis and HPLC and eliminates the need for a laboratory. Gazelle yielded an overall high accuracy (99.0%) compared to reference standard tests (HPLC and hemoglobin electrophoresis). These results were consistent with the accuracy of a previously described prototype version of Gazelle, HemeChip (16). The overall diagnostic accuracy of HemeChip was 98.4% in identifying SCD-SS, SCD-SC, SCD Trait, Hb E Disease, Hb E Trait, and Normal (16). Although, Gazelle showed an accuracy of 93.5% in additional analysis wherein inconclusive test results were considered are incorrect, these results represent a hypothetical condition. In the real-world setting, an inconclusive test results would not yield a wrong diagnosis as Gazelle prompts a retest. Two Beta thalassemia major subjects were incorrectly identified as Hb SS disease by Gazelle which is a limitation of the present algorithm, but its capacity to identify Beta thalassemia as “disease” is useful. Furthermore, it would be beneficial to test more samples with high HbF and relatively lower HbA or HbS for improving the current diagnostic algorithm for accurate detection of beta thalassemia and other variants. It may be noted that the current study recruited subjects only between the ages of 6 months to 85 years as per inclusion criteria. In all only 2 infants of age 1 year were recruited. No newborn or infant <6 months were included in the study as current version was not suitable for newborn screening. The improved version of “Gazelle” with multispectral imaging and newer algorithm and software has been found suitable for newborn screening (Personal Communication).

Gazelle offers the benefit of both standard hemoglobin electrophoresis and a point-of-care test. Gazelle replaces the bench top laboratory setup with a portable reader and replaces the hemoglobin controls with a blue control marker (19, 20). On an average it took about 13 min from drawing of blood sample to completing the run on Gazelle. The entire process was about 8 times faster than hemoglobin electrophoresis (~2–3 h) and comparable to HPLC (~10 min), however, as HPLC is run as a batch, the time from sample draw to result was much longer. Gazelle provides hemoglobin type identification and quantification simultaneously and offers a complete interpretation of test results, thus avoiding transcription and interpretation errors. The current study showed that identification of various hemoglobin variants by Gazelle was equivalent to those obtained in hemoglobin electrophoresis or HPLC. Furthermore, hemoglobin electrophoresis is labor intensive, time consuming and laboratory dependent compared to HPLC which is also cost intensive and requires a laboratory setting.

To counter high cost burden of advanced laboratories, a need for a low-cost reliable point of-care test with high accuracy and specificity is needed. In addition to clinical benefits, economic benefit has been one of the primary objectives of Gazelle design (16).

These are several features of Gazelle that makes it a potential diagnostic tool for field use in detection and management of SCA endemic areas of the world. Gazelle is a rugged, battery-operated cartridge-based point-of-care, sickle diagnostic device that is easy to use and quickly provides accurate results in the field. The quick turnaround times and low-cost test can enable clinicians to perform widespread screening affordably and quickly identify carriers of sickle hemoglobin. Gazelle provides easily readable qualitative as well as quantitative results that are electronically stored and can be transmitted. One other Gazelle feature that limits user errors and the time needed for training is the included step by step instructions displayed on the screen to guide the user during the testing process. Overall, this study adds to the increasing evidence that microfluidic electrophoresis assays may be a valuable tool for early identification of affected population resulting in better clinical management and reduction in morbidity and mortality due to SCD. Gazelle has a high sensitivity and specificity in detection of SCD and SCD trait. Diagnostic accuracy of Gazelle is comparable to conventional hemoglobin electrophoresis testing. Gazelle's low cost, need for minimal training, portability, and fast test turnaround time makes it ideally suited for low-resource settings, wherein Gazelle can facilitate quick diagnosis and early clinical management of hemoglobin disorders. Future work on extending the test to newborns and identifying additional hemoglobin variants would be beneficial.

## Data Availability Statement

The original contributions presented in the study are included in the article/supplementary material, further inquiries can be directed to the corresponding authors.

## Ethics Statement

The studies involving human participants were reviewed and approved by Institutional Ethical Committee of ICMR-National Institute of Reaserch in Tribal Health, Jabalpur, M.P., India. Written informed consent to participate in this study was provided by the participants' legal guardian/next of kin.

## Author Contributions

AD, PT, RS, and PB designed the study and critically reviewed the manuscript. MP, RK, AV, SS, RU, and ST collected the samples and executed the experiments. PT, RS, PB, and AD provided resources and infrastructure for the study. AV, AG, RK, SS, and PB analyzed the data. SS, AV, PT, PB, and RS wrote the manuscript. All authors read and approved the manuscript.

## Funding

RS reports grants and non-financial support from Hemex Health, USA. Grant Number: GZL-M10-001.

## Conflict of Interest

RS reports grants and non-financial support from Hemex Health, USA. PT is an employee of Hemex Health USA. The remaining authors declare that the research was conducted in the absence of any commercial or financial relationships that could be construed as a potential conflict of interest. The reviewer SSM declared a shared affiliation with several of the authors SS, MP, RK, AG, RU, ST, AV, AD, PB, and RS to the handling editor at time of review.

## Publisher's Note

All claims expressed in this article are solely those of the authors and do not necessarily represent those of their affiliated organizations, or those of the publisher, the editors and the reviewers. Any product that may be evaluated in this article, or claim that may be made by its manufacturer, is not guaranteed or endorsed by the publisher.
